# Evaluation of the physicochemical and functional stability of diluted *REMSIMA*^®^ upon extended storage—A study compliant with NHS (UK) guidance

**DOI:** 10.1016/j.ijpharm.2015.10.016

**Published:** 2015-12-30

**Authors:** Benjamin L. Young, Monika Ali Khan, Terry J. Chapman, Richard Parry, Maria A. Connolly, Andrew G. Watts

**Affiliations:** a Department of Pharmacy & Pharmacology, University of Bath, Claverton Down, Bath BA2 7AY, United Kingdom; b Bath ASU, Unit A15 Fiveways Light Industrial Estate, Westwells Road, Corsham SN13 9RG, United Kingdom

**Keywords:** Remsima, Infliximab, Biosimilar, Antibody, Shelf-life, Extended storage, Compliance, Stability study

## Abstract

A newly licensed biosimilar product containing infliximab as the active pharmaceutical ingredient has recently been marketed under the brand name Remsima^®^. We have evaluated the stability of Remsima^®^ diluted in sodium chloride solution and stored in polyolefin bags at 2–8 °C using a range of techniques to assess the physico-chemical and functional integrity of the drug over time. The methods and techniques employed are fully compliant with NHS (UK) guidance for evaluating the stability of biologicals, enabling the data to be used for the application of an extended shelf-life to Remsima products in the UK, when prepared under a Section 10 exemption or a Specials Licence. The results clearly demonstrate physico-chemical and functional stability of the drug over the 7 day period of the study, when prepared as described here under aseptic conditions in accordance with the Summary of manufacturers Product Characteristics.

## Graphical abstract

**Figure:**
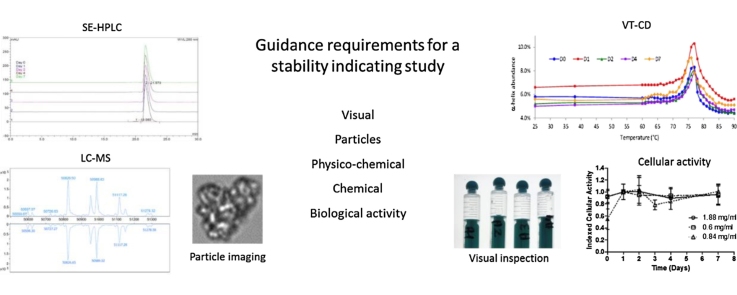

## 1 Introduction

Infliximab (originally marketed as *Remicade*^®^ by Merck, Sharp and Dohme) is a chimeric human-murine IgG_1_ monoclonal antibody (mAb) which acts as a cytokine modulator (Knight et al., 1993). It works by selectively binding to soluble and transmembrane forms of TNF-α, thereby reducing the pro-inflammatory signalling of this cytokine (Wong et al., 2008, Couriel et al., 2000). Infliximab is indicated for the treatment of rheumatoid arthritis (in combination with methotrexate) when treatment with other anti-rheumatic drugs have failed, and for ankylosing spondylitis. Dosing for all indications is according to body mass and ranges from 3 to 7.5 mg/kg (Electronic Medicines Compendium, 2015a). The recent patent expiry of *Remicade*^®^ has resulted in the emergence of biosimilar versions of infliximab, one of which is *Remsima*^®^, marketed in the United Kingdom by NAPP Pharmaceuticals. The pharmaceutical form, strength, size and composition of this medicinal product is reported to be the same as that of the *Remicade*^®^ brand, according to the relevant Summary of manufacturers Product Characteristics (SmPC) (Electronic Medicines Compendium, 2015a, Electronic Medicines Compendium, 2015b).

The manufacturers of both *Remicade*^®^ and *Remsima*^®^ have stated that the medicinal product is physico-chemically stable for up to 24 h when stored at 25 °C and once prepared, storage should not exceed 24 h at 2–8 °C. Such short shelf-lives are not uncommon for this class of product, with the vast majority of antibody therapeutics being assigned storage limits of either 24 or 48 h, once prepared in ready to use form. However, it is commonly considered that these short shelf-lives are applied to limit any potential risk from microbiological contamination introduced during preparation, as opposed to them reflecting an inherent lack of physico-chemical stability of the drug.

In the United Kingdom, extended shelf-lives can be applied to antibody therapeutics prepared under a Section 10 exemption or a Specials Licence, where robust data exists to support them. Here, the National Health Service (NHS) Guidance document ‘*A Standard Protocol for Deriving and Assessment of Stability; Part 2: Aseptic Preparations (Biopharmaceuticals). Edition 1, October 2012*’, serves to define the criteria necessary for a robust study of stability, towards the application of an extended shelf-life to the clinical product (Santillo, 2012).

Herein, we report on the stability profile of diluted *Remsima*^®^ stored over a 7 day period in ready to use form at a range of clinically relevant concentrations. Furthermore, the testing methods and techniques employed here are fully compliant with guidance criteria set out by the NHS (UK) and, as such, constitute a reliable study for the application of an extended shelf-life to the clinical product when prepared under Section 10 exemption or a Specials Licence. To the best of our knowledge, this report represents the only published study currently compliant with national guidelines in the UK to apply an extended shelf-life to this class of product.

## 2 Study design

Guidance provided by the NHS (UK) was used as a template for both the design of study methods, as well as the combination of analytical techniques employed (Santillo, 2012). All guidance requirements for the design of a robust stability study are detailed in Table 1, along with the elements employed in this study.

### 2.1 Study elements

Polyolefin (*Freeflex*^®^) infusion bags were chosen as storage containers and all drug samples were diluted using 0.9% sodium chloride solution.

It is required by guidance that both clinically low and high concentrations be studied, so that if the drug shows a consistent stability profile it should be possible to interpolate stability between the concentrations studied. However, given the complexity and inherent variability of this drug class, it was decided that the study should exceed guidance requirements and three concentrations of drug needed to be studied here. As such, this study evaluated clinically low (0.60 mg/mL), clinically high (1.88 mg/mL) and clinically common (0.84 mg/mL) concentrations of drug.

It is recommended that two storage conditions are employed, refrigerated (2–8 °C) for the period of the study and room temperature (25 °C) for 24 h. This study evaluated all three concentrations of drug under refrigerated storage (in the absence of light) for the period of the study, as well as the clinically common concentration (0.84 mg/mL) stored at 25 °C for 24 h (exposed to light) to represent in-use conditions.

A study period of 7 days was chosen so that the study was applicable to the data but as short as practicable. Sampling strategy was designed to include the required number of sampling points for a 7 day study, those being four sampling points in addition to baseline (*T* = 0) data.

For adequate sampling numbers, three independent batches are required, with each batch needing to be evaluated in triplicate. Again, given the complexity and inherent variability of this drug class, it was decided that the study should evaluate more replicate devices within each batch. As such, four independent replicate devices were prepared for each of the three batches (one batch at each concentration of drug), meaning that a total of 12 individual storage containers were prepared. Additionally, to perform a truly representative evaluation of batch-to-batch variation during manufacturing, the batch of drugs at clinically common concentration (0.84 mg/mL) were prepared (and subsequently analysed) two weeks apart from the batches of clinically low and high concentrations.

### 2.2 Test methodology

Biopharmaceuticals degrade through a wide variety of pathways and, as such, a range of analytical techniques are required to adequately evaluate the physical, chemical and functional integrity of these molecules when stored for extended periods of time. NHS guidance provides a series of criteria that must be addressed for the evaluation of stability to be considered robust (Table 2) (Santillo, 2012).

Forced degradation studies need to be performed to demonstrate the stability indicating nature of the analysis methods employed. The stress conditions employed here to degrade Remsima were chosen on the basis that they represent the most likely forms of stress to be encountered during use. As such, degradation resulting from changes to pH (high and low), oxidation, or exposure to light were demonstrated at room temperature for all appropriate analysis methods (online Supplemental data).

The visual characteristics of all test samples were evaluated to detect gross physical changes to the drug by inspection of colour, clarity and presence of visible particulates.

The stability of monoclonal antibodies is highly dependent on the pH of the solution, which may change over time due to interactions with excipients, infusion bag materials or as a result of protein degradation. As such, the pH of drug solution was evaluated in triplicate for all test samples.

The presence of sub-visible particles over the range 1–100 μm was evaluated by particle imaging (FlowCam^®^, Fluid Imaging Technologies) as this technique provides several advantages to alternative methods, such as turbidity measurements. In particular, particle imaging provides a quantitative assessment of particle numbers, as well as the ability to characterise the type of particle detected (i.e. silicon oil droplet, micro-air bubble, protein) which can provide a more detailed understanding of degradation.

Evaluation of physico-chemical stability needs to assess multiple structural and chemical aspects of the protein, requiring a combination of several analytical techniques to be performed. Here, size exclusion chromatography (SEC) and gel electrophoresis (Tapestation 2200, Agilent) were used to identify significant changes in protein molecular weight, such as reversible protein aggregation (dimerisation and trimerisation) or hydrolysis. Dynamic light scattering (DLS) was used to evaluate levels of aggregates and particles over the range 10 nm–6 μm (percentage abundance) as well as gross changes to tertiary and quaternary structure (hydrodynamic radius). Variable temperature circular dichroism (VT-CD) evaluated changes to secondary and tertiary structure of the protein by determining percentage composition of α-helices and β-sheets, while melting temperature (*T*_m_) provides an indication of changes to the chemical bonding (hydrogen bonding, disulphide bridges) contributing to the stability of these structures.

In-line HPLC-mass spectrometry was used to provide a comprehensive evaluation of chemical changes to the antibody. This technique was chosen as any observed mass differences can be used to characterise specific chemical modifications occurring, such as deamination, oxidation, deglycosylation and hydrolysis.

For the evaluation of changes to functional activity, a cell based assay based on the procedure of Espevik and Nissen-Meyer was chosen (Espevik and Nissen-Meyer, 1986). This method provides a direct measure of the biological activity responsible for the therapeutic effect of the drug, as required by NHS guidance (Santillo, 2012).

## 3 Materials and methods

### 3.1 Reagents

Sodium phosphate monobasic, sodium phosphate dibasic dihydrate and sodium chloride were obtained from Sigma–Aldrich (Gillingham, Dorset, UK) and were of Analytical Reagent or HPLC Grade. Water used for the preparation of buffers and other analytical solutions was of HPLC Grade (*Chromasolv*^®^) and obtained from Sigma–Aldrich. Water for injections was obtained from *Baxter Ltd.*^®^ (Thetford, Cambridgeshire. UK). RPMI 1640, heat inactivated foetal bovine serum (FBS), Penicillin/Streptomycin, Phosphate Buffered Saline (PBS), HyQtase and MTT were all purchased from Gibco. Tris(3-hydroxypropyl) phosphine (THPP) was purchased from Sigma–Aldrich.

### 3.2 Materials

*Remsima*^®^ 100 mg vials (BN: 14B1M016DC1, expiry 03/2019) were obtained from NAPP Pharmaceuticals (Thetford, Cambridgeshire, UK). *Freeflex*^®^ infusion bags of sodium chloride 0.9% sterile solution for infusion were used for storage and sample dilution and were obtained from *Fresenius Kabi* (Runcorn, Cheshire, UK). Lubricant-free luer-lock Injekt® syringes (BN: 4606728V) were obtained from *B. Braun* (Melsungen, Germany). WEHI 164 cells (ATCC; CRL-1751) were purchased from ATCC. Corning T75 tissue culture treated flasks and Nunc TC-treated Flat bottomed 96 well plates were purchased from Fisher Scientific. TNF-α and Actinomycin D were purchased from RnD Systems.

### 3.3 Methods

*Remsima*^®^ products under study were prepared under aseptic conditions in line with Good Manufacturing Practice, using validated pharmacy procedures and in full accordance with instructions outlined within the Summary of manufacturers Product Characteristics for *Remsima*^®^ (Electronic Medicines Compendium, 2015a). Products were prepared at three concentrations of 1.88 mg/mL, 0.84 mg/mL and 0.60 mg/mL by reconstituting the vials with water for injection to give a 10 mL concentrated stock solution. The stock solution was added to a *Freeflex*^®^ polyolefin infusion bag of sodium chloride 0.9% and stored at 2–8 °C in light-protective bags. On the day of analysis, samples were removed from each test product bag using aseptic technique by withdrawing 4–5 mL into a lubricant-free syringe, which was capped and stored at 2–8 °C during the analysis period. All test products were prepared in quadruplicate thus a total of 4 bags at 1.88 mg/mL, 4 bags at 0.84 mg/mL and 4 bags at 0.60 mg/mL were compounded and used for analysis.

Samples were analysed on the day of test product preparation (Day 0) and then at days 1, 2, 4 and 7 for batches at 1.88 mg/mL and 0.60 mg/mL. Samples were analysed on the day of test product preparation (Day 0) and then at days 1, 2, 3 and 7 for the batch at 0.84 mg/mL. Test product samples were analysed using the listed range of physico-chemical and biological techniques to assess the physical, chemical and functional stability profiles of *Remsima*^®^ at each time point.

### 3.4 Forced degradation

The stability indicating nature of each method was demonstrated through forced degradation studies (Supplemental online data). A range of degradation pathways were assessed by subjecting freshly prepared samples of diluted *Remsima*^®^ (1.5 mg/mL in 0.9% NaCl solution) to several stress conditions which included low pH (10 mM HCl), high pH (10 mM NaOH), oxidation (2% hydrogen peroxide) and exposure to fluorescent light (Manning et al., 2010). All samples were analysed immediately following stress.

### 3.5 Visual characteristics

Prior to any analytical methods being performed, samples were checked by the unaided eye under normal laboratory fluorescent light for evidence of particulates, precipitation, colour change and/or turbidity.

### 3.6 pH

Samples were tested using a *Jenway 3510* pH Meter and 924007 electrode (Bibby Scientific, Staffordshire, UK) which was calibrated prior to use at pH 4.00, 7.00 and 14.00. Triplicate pH measurements were taken for each sample tested and results are presented as the mean average of the triplicate measurements.

### 3.7 Sub-visible particle counting

Particle counting was performed using a *FlowCAM VS* instrument (Fluid Imaging Technologies Inc., MA, USA) fitted with an *FC100FV* flow cell and able to detect and quantify particles of 1–100 μm in size. Flow rate was set to run at 0.15 mL/min, with an imaging rate of 20 frames per second and an efficiency of 30.2%. System calibration was performed using 10 μm polysorbate beads (Sigma–Aldrich). Data was analysed using *Visual Spreadsheet* software (v. 3.0.3). The stability indicating nature of this method was confirmed through forced degradation studies.

### 3.8 Physico-chemical analysis

#### 3.8.1 SE-HPLC

Size exclusion HPLC as performed on an *Ultimate 3000* system (Dionex, Sunnyvale, CA, USA), consisting of an LPG-3400BM pump, a WPS-3000TBFC analytical autosampler, a TCC-3000SD column oven and a VWD-3100 detector. The samples were eluted on a *TSKGel*^®^ G4000SW_XL_ column (TOSOH BioScience, Stuttgart, Germany) with dimensions 7.8 × 300 mm and particle size 8 μm 450 Å. The mobile phase was 300 mM NaCl + 50 mM Na_3_PO_4_ buffer at pH 6.8 in HPLC grade water. The injection volume was 20 μL, with an isocratic flow rate of 0.5 mL/min over 30 min and UV absorbance measured at 280 nm. Data was acquired and analysed using Dionex^®^ *Chromeleon* software (v 6.8). Drug concentrations were obtained from peak signal area by the use of a linear regression curve of *Remsima*^®^ concentration vs absorption. Full validation of the HPLC method was performed. This included demonstration of accuracy, precision and linearity over the range of concentrations tested, as well as demonstrating that limits of detection (LOD) and quantification (LOQ) were sufficient for robust evaluation of protein characteristics (online Supplemental data). The stability indicating nature of this method was confirmed through forced degradation studies.

#### 3.8.2 Dynamic light scattering

Samples were analysed undiluted on a *Zetasizer Nano*-*S* instrument at 20 °C (Malvern Instruments., Worcs., UK) using a red laser at a wavelength of 633 nm and a Hellma Quartz-Suprasil cuvette Type 105.251.005-QS. The light path and centre were set at 3 × 3 mm and 9.65 mm, respectively. All data was recorded based on intensity and converted to relative percentage by volume using *Zetasizer* software v.6.20 and cumulants fit analysis. The stability indicating nature of this method was confirmed through forced degradation studies.

#### 3.8.3 Gel electrophoresis

Protein separation analysis was conducted using samples which were diluted to a concentration of 0.5 mg/mL with HPLC grade water. Samples were analysed with the Screen Tape P200 Protein Standard Kit (Part number: 5067–5371, Agilent) under reducing and non-reducing conditions using P200 Reagents (Part number: 5067–5372) on a 2200 Tape Station system (Agilent^®^ Technologies, Waldbronn, Germany) according to the manufacturer's instructions. The “P200 Markers (pre-stained)” (Agilent) was used as a molecular marker. The stability indicating nature of this method was confirmed through forced degradation studies.

#### 3.8.4 Variable temperature circular dichroism

Variable temperature Circular Dichroism was performed using a *Chirascan* spectrophotometer (Applied Photophysics Ltd., Surrey, UK). Samples from each test product bag were pooled and diluted to a concentration of 0.25–0.3 mg/mL with sodium chloride 0.9% and tested using a Quartz Suprasil Cuvette (Hellma Analytics, Essex, UK) of pathlength 0.1 cm. CD spectra were collected in the far-UV region (205–260 nm) over a temperature range of 25–90 °C in steps of +1 °C/min. Final CD spectra were adjusted against the blank (NaCl 0.9%) and analysed using CDNN deconvolution software (v2.1) to yield composition percentages of secondary structures identified as of alpha-helices, parallel, anti-parallel, beta-turn and random coil structure. The stability indicating nature of this method was confirmed through forced degradation studies.

#### 3.8.5 LC mass spectroscopy

Mass spectroscopy was conducted using a 6520 Accurate-Mass Q-TOF mass analyser instrument (Agilent^®^, Germany). Samples were desalted by centrifugation at 4000 rpm for 30 min and rinsing with HPLC Grade Water. This process being repeated 4 times to ensure complete salt removal and therefore avoiding interference of ionisation by the presence of sodium chloride. Samples of 0.5 mL were then reduced by adding 5 molar equivalents of THPP. This also allows for the generation of separate spectra profiling ions which are derived from both the heavy and light chain. The stability indicating nature of this method was confirmed through forced degradation studies.

### 3.9 Biological activity

The biological activity of *Remsima*^®^ samples was assessed by their ability to inhibit TNF-α induced cell death in the WEHI cell line following the procedure of Espevik and Nissen-Meyer (Espevik and Nissen-Meyer, 1986). Cells were harvested from culture and added to a 96 well plate at 1.5 × 10^4^ cells/well and incubated for 2  at 37 °C for the cells to adhere. Remsima samples were diluted to 1 μg/mL in saline and incubated with 10 pg/mL TNF-α for 30 min. The TNF-α antibody mixture was then added to the cells and 2 μg/mL Actinomycin D added to the wells. Assay plates were incubated for a further 18 h at 37 °C. Cell viability was then assessed using an MTT assay. To analyse the results, the absorbance at 540 nm (A540) of cells exposed to TNF-α was set as background and subtracted from all values. A540 of cells not exposed to TNF-α (negative control) was set to 100%. Percent cell viability was then calculated as (A540 sample/A540 negative control) × 100. Values were then indexed against values obtained for day 0 vial samples of infliximab to control for inter-assay variability. Each sample was tested in quintuplicate and the mean and standard deviation calculated. The stability indicating nature of this assay method was confirmed through forced degradation studies.

## 4 Results

### 4.1 Visual inspection

All samples remained clear for the duration of the study with no precipitates or particulate matter detected with the naked eye. No change in colour or turbidity was observed over the study period. Photographs of all storage devices tested at each time point are provided in the online Supplemental data.

### 4.2 pH

**Fig. 1:**
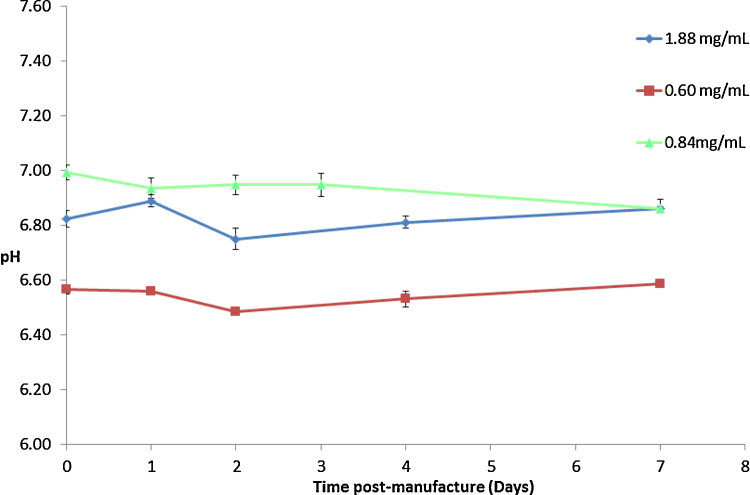
Mean pH profile of *Remsima*^®^ for 0.60 mg/mL, 0.84 mg/mL and 1.88 mg/mL solutions over the study period (*n* = 4). Error bars represent ± 1 SD.

No significant change was observed for the pH of samples at any of the concentrations tested throughout the study period (Fig. 1). The pH of samples at 1.88 mg/mL and 0.84 mg/mL were very similar at around pH 6.9, while the pH of the most dilute samples at 0.60 mg/mL was noticeably lower at pH 6.6, possibly reflecting the buffering capacity of excipients. Slight variability in pH readings (±0.1 pH units) was observed for all concentrations, however this variability was not significant as the observed pH’s at day 7 showed almost no change from their respective pH’s at day 0, for all concentrations.

### 4.3 Sub-visible particle counting

**Fig. 2:**
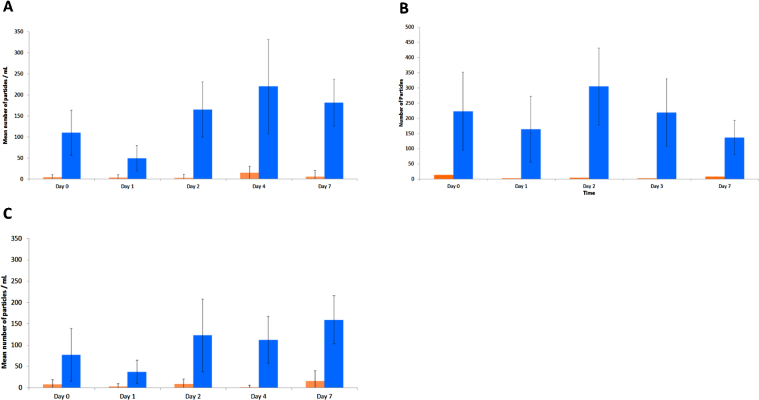
Mean particle number results from FlowCam imaging of Remsima^®^ at (A) 0.60 mg/mL, (B) 0.84 mg/mL and (C) 1.88 mg/mL, over the period of the study. Particles >10 μm are shown in blue and particles > 25 μm are shown in orange. Error bars represent ± 1 SD.

Quantification of particle numbers was carried for particles >10 μm and >25 μm in size. At all concentrations tested, the number of particles >10 μm was significantly higher than the number of particles >25 μm for all time points. Also, as is common with flow imaging techniques, the standard deviations for replicate samples was found to be quite large, making a detailed statistical analysis of results difficult. However, for all concentrations tested the largest variations in particle numbers appeared to be between the early time points (day 0, 1 and 2), with particle numbers appearing to remain relatively constant past day 2. Importantly, there appeared to be no appreciable change in average particle numbers between samples at day 0 and day 7, for any of the concentrations tested Fig. 2.

### 4.4 Size-exclusion HPLC

**Fig. 3:**
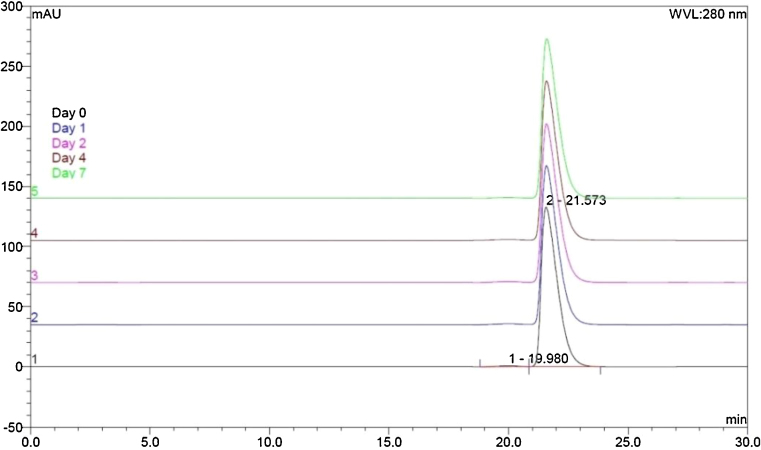
Representative overlay of SE-HPLC chromatograms of Remsima (1.88 mg/mL) at Days 0, 1, 2, 4, and 7. Samples at each time point were run in triplicate and an average peak-signal calculated.

All HPLC chromatograms of Remsima^®^ were characterised by a single major peak with an elution time of 21.6 min (Fig. 3). For the clinically high concentration batch (1.88 mg/mL) this comprised, on average, 99.28% (±0.15%) of all detected signals over the course of the study. A minor signal with a retention time around 20.0 min was also observed at this concentration, which comprised the remaining 0.73% of observed signals. This minor peak was assigned as Remsima dimer on the basis of the retention time equating to a molecular weight of 300 kDa, according to a standard protein curve (online Supplemental data). Chromatograms obtained from samples at 0.84 mg/mL and 0.60 mg/mL comprised of only a single peak corresponding to the Remsima monomer.

Importantly, no change in the intensity of signals was observed for any of the batches over the period of the study, indicating that the concentration of Remsima remained constant throughout (Table 3).

### 4.5 Variable temperature CD

**Fig. 4:**
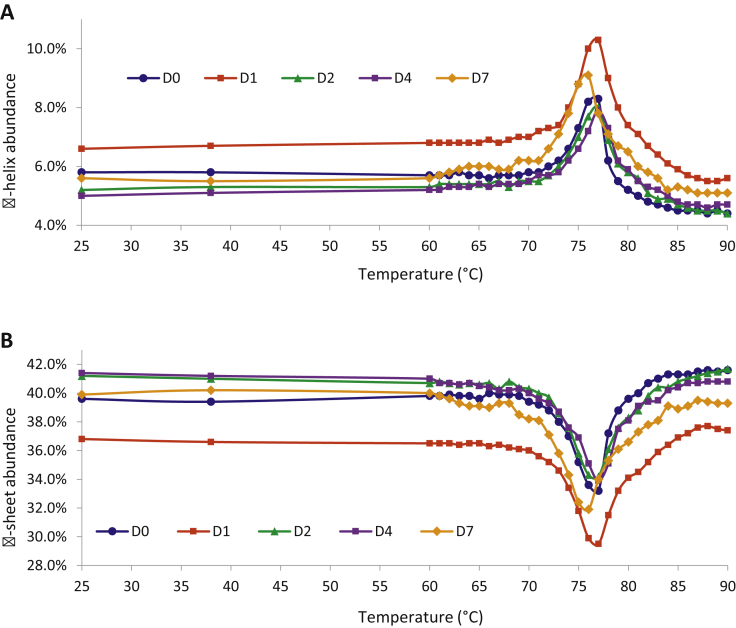
Variable temperature circular dichroism data for Remsima^®^ samples at 1.88 mg/mL showing structural abundance of (A) α-helix, and (B) β-sheet as a percentage of the total protein. Measured over a temperature range of 25 to 90 °C.

Analysis of variable temperature circular dichroism spectra indicates the protein undergoes its most significant changes to secondary structure induced by thermal degradation over the temperature range 74–78 °C (Fig. 4). This temperature range remained largely constant throughout the study period for all concentrations tested.

The percentage composition of secondary structures (α-helix and β-sheet) measured at 76 °C was found to vary slightly over the course of the study, though no appreciable changes were observed. For the more concentrated samples (1.88 mg/mL), α-helix content varied between 7.2% and 10% during the study, while β-sheet varied between 29.9% and 35.1%. At this concentration, the samples at day 1 showed the greatest deviation from results at other time points whilst, importantly, day 7 data fell between the range of the early time points (day 0 and day 1) Table 4.

For the more diluted samples (0.60 mg/mL), slightly less variation in secondary structure was observed, with α-helix content varying between 8.3% and 9.7% during the study, while β-sheet varied between 30.7% and 33.2%. At this concentration it was the day 2 samples that showed the greatest deviation from results at other time points, indicating that changes to secondary structure may be occurring slower than for the higher concentration samples. Additionally, for both the high and low concentrations, it is the early time points in the study that exhibit the greatest degree of variations in secondary structure.

### 4.6 Dynamic light scattering

**Fig. 5:**
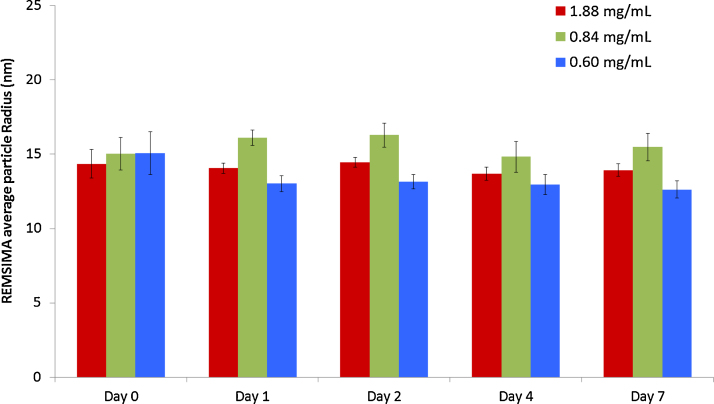
Averaged particle radius of 1.88 mg/mL, 0.84 mg/mL and 0.60 mg/mL Remsima^®^ samples over the course of 7 days as determined by Dynamic Light Scattering. Each time point consisted of 4 different samples at each concentration, which in turn were run in triplicate to provide an average measurement for each bag (*n* = 12).

Dynamic light scattering results are expressed as the mean radius and percentage abundance of detected drug particles within the test solution (Fig. 5). A significantly larger mean radius could suggest the formation of aggregates which have a greater spherical volume, while a decrease in percentage abundance of drug could be attributed to larger aggregate formation. The measured drug radii were found to be similar for all concentrations throughout the study, ranging from 14.3 to 15.1 nm immediately after preparation on day 0, to 12.9–15.2 nm on day 7.

Additionally, the percentage abundance of drug particles remained constant over the study period, demonstrating that there was no significant increase in the number of larger particles (<6 μm) at any of the concentrations (Table 5).

### 4.7 SDS gel electrophoresis

**Fig. 6:**
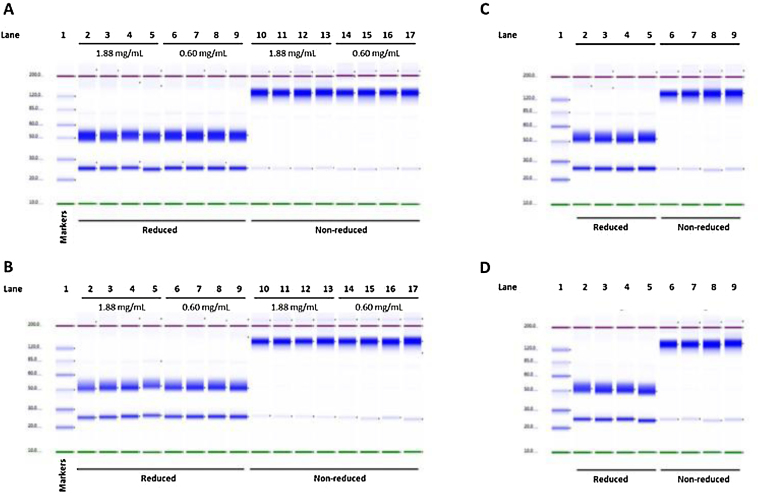
Protein separation analysis of and Remsima^®^ samples (5 μg per well) showing (A) Day 0 for 1.88 mg/mL and 0.60 mg/mL, (B) Day 7 for 1.88 mg/mL and 0.60 mg/mL, (C) Day 0 for 0.84 mg/mL and (D) Day 7 for 0.84 mg/mL. Lane 1 in all gels is a protein ladder standard.

Protein separation of day 0 samples for all concentrations was characterised by a single band of approximately 150 kDa for the native (non-reduced) samples and two bands of approximately 50 kDa and 25 kDa for the reduced samples (Fig. 6A and C). No change to either the molecular weight or intensity of the bands was observed for any samples throughout the study period for all concentrations, as is demonstrated by the day 7 samples shown (Fig. 6B and D).

### 4.8 LC mass spectroscopy

**Fig. 7:**
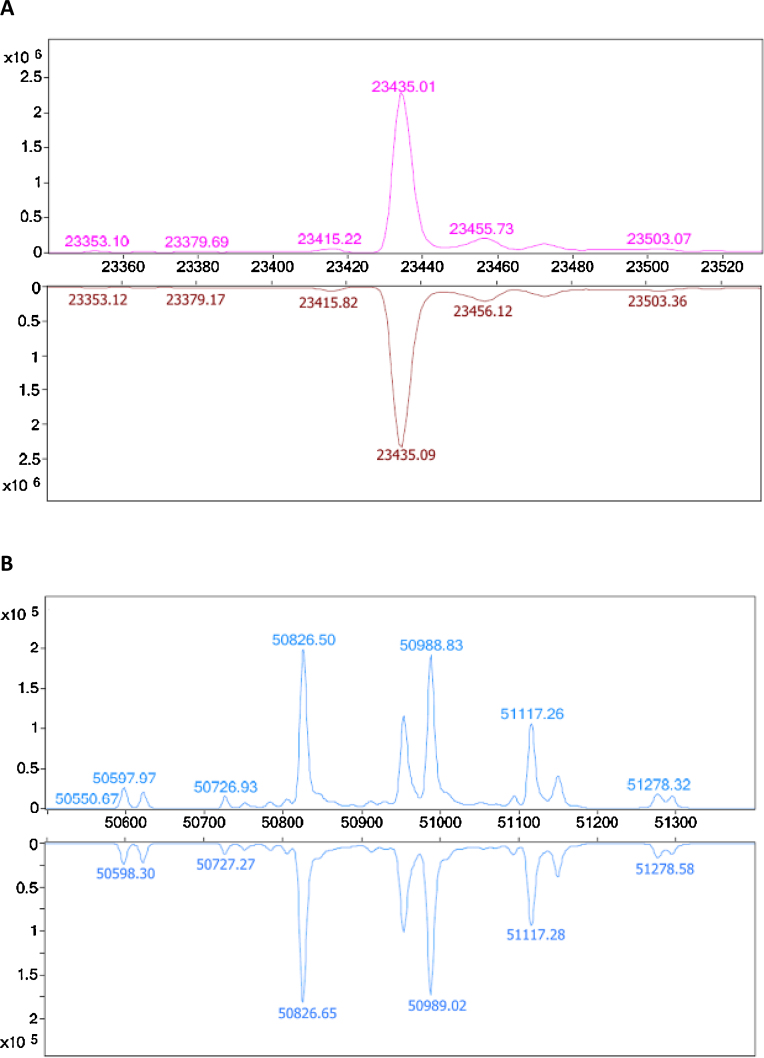
Comparison of LC–MS spectra for samples of Remsima stored at 0.60 mg/mL. Day 0 (top) vs Day 7 (bottom) spectra are compared for (A) light chain and (B) heavy chain.

Mass spectral analysis of the light chain of Remsima on day 0 across all concentrations is characterised by a single major species of mass 23.4 kDa (Fig. 7A). The mass of this species remained constant over the study period, with no additional peaks appearing, indicating that the chemical identity of the light chain did not change (online Supplemental data).

The mass spectrum of the heavy chain of Remsima is characterised by a series of molecular ion peaks of around 51 kDa in mass (Fig. 7B). The complexity of this spectra is characteristic of antibody heavy chains owing to the heterogeneity of *N*-linked glycan profiles. Comparison of the heavy chain mass spectra at day 0 and day 7 (for all concentrations) revealed no obvious change in chemical composition at any point during the study period. Importantly, this technique demonstrates the stability of the primary peptide chain, as well as the glycan profile of the antibody which can influence functional activity and pharmacokinetics of the protein.

### 4.9 Biological activity

**Fig. 8:**
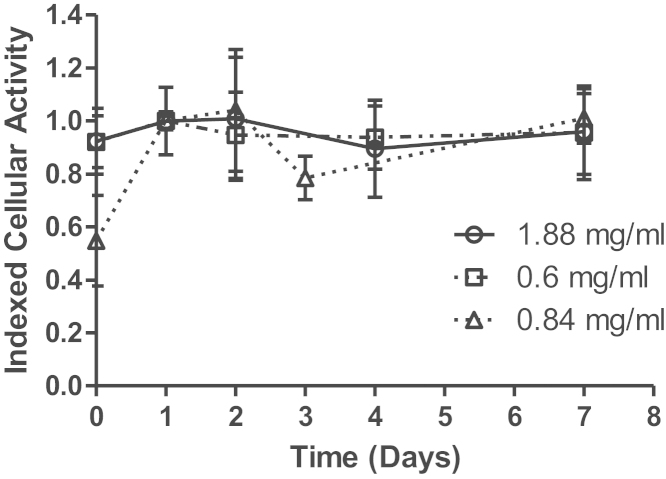
Functional activity of Remsima was tested by its ability to neutralise TNF-α in a WEHI cell death assay. Remsima was tested at 1 μg/mL and results indexed against results for Remicade to control for inter assay variation. Data shown are mean with SD of pooled results for each concentration.

Samples of diluted Remsima typically prevented around 80% of the TNF-α induced death of WEHI cells when tested at 1 μg/mL in this assay. This level of biological activity was equivalent to that observed for freshly prepared samples of Remicade (1 μg/mL). Samples of Remsima tested at Day 0 showed the most dramatic variability in biological activity, both across the three concentrations (batches) and within each batch (Fig. 8). This variability in activity appeared to diminish over the period of the study, such that Day 7 data demonstrated the least amount of inter- and intra-batch variability. Owing to the variability in the data, statistical analysis (*t*-test) was performed to identify changes of significance in the biological activity of the drug. Day 1 (SmPC limit) data was compared to both Day 0 and Day 7 data, with results presented in Table 6. Interestingly, analysis demonstrated a statistically significance in biological activity between Day 0 and Day 1 samples (within the SmPC assigned shelf-life) at all concentrations tested. However, no significance was observed to differences between activities at Day 1 and Day 7, across all concentrations.

## 5 Discussion

This study has employed study design and testing methodology which are compliant with NHS guidance requirements for performing a robust evaluation of antibody stability over a period of extended storage. We have used a number of analytical and biological techniques to evaluate the stability of diluted solutions of Remsima for a period of 7 days. Overall, no chemical or physical instability was observed, while the drug was also found to retain full biological activity.

The techniques of LC–MS, SE-HPLC and gel electrophoresis showed no change for any sample examined throughout the study, indicating that full integrity of the chemical structure (protein and carbohydrate) of the drug is retained.

However, variable temperature circular dichroism does indicate minor changes in the secondary structure of the drug immediately following dilution, which appears to be accompanied by variability in numbers of particles >10 μm in size. These observations are not surprising, as dilution of the excipients results in a dramatic reorganisation of protein surface interactions. In all cases however, the observed variability is reduced over time as the drug gradually reaches equilibrium under new conditions.

It is also of interest to note that, although minor, the initial changes to secondary structure and variability in particle number do appear to show a measurable impact upon the biological activity of the drug. A statistically significant difference can be attributed to the mean activities of day 0 and day 1 samples, whilst no such difference is present between day 1 and day 7 samples. In totality, these results suggest that the process of drug dilution contributes a more dramatic impact on drug quality than does extended storage, at least over the storage period studied here.

## 6 Conclusions

Here we have demonstrated the stability of Remsima^®^ over a 7 day period when diluted in sodium chloride solution and stored in polyolefin bags at 2–8 °C. A range of analytical techniques were used to assess the physico-chemical and functional integrity of the drug over time. Furthermore, the methods and techniques used here are fully compliant with NHS (UK) guidance for evaluating the stability of biologicals, enabling this data to be used for the application of 7 day shelf-life to Remsima products, when prepared under a Section 10 exemption or a Specials License and according to the methods presented here.

## Conflict of interest

A.G. Watts performs consulting activities for Bath ASU.

## Figures and Tables

**Table 1:** Guidance requirements for design of a robust stability study, with the elements employed in this study listed alongside to demonstrate compliance (Santillo, 2012).

Study element	Guidance requirement	Element employed in this study
Diluent	As specified in SmPC	0.9% sodium chloride solution
Containers	Non-PVC	Polyolefin (Freeflex^®^)
Storage	Refrigerated in absence of light	Refrigerated in absence of light
Room temperature (25 ± 2 °C)	Room temperature (24 h) for clinically common (0.84 mg/mL)
Concentrations	Low & high clinically significant	Three concentrations tested. Clinically low (0.60 mg/mL), clinically common (0.84 mg/mL) and clinically high (1.88 mg/mL)
Storage period	Normally 48 h–3 months	Seven day storage
Sampling strategy	For studies <6 months, at least 4 sampling points in addition to the baseline (*T* = 0) data.	Sampling performed at day = 0, 1, 2, 4 and 7 for low/high conc.
Sampling performed at day = 0, 1, 2, 3 and 7 for common conc.
Sampling number	Three independent batches	Three independent batches, one batch at each concentration
Three replicates per batch	Four independent replicate containers within each batch

**Table 2:** NHS guidance requirements for a robust testing methodology and the methods employed in this study.

Test methodology	Guidance recommendation	Methodology employed in this study
Forced degradation	A combination of some of:	Impact of acid, base, oxidation and exposure to light (natural and fluorescent) were evaluated for each technique
(a) Change in pH
(b) Realistic elevated temperature
(c) Exposure to UV light
(d) Agitation
Visual characteristics	Colour, clarity and particulates	Visual inspection of colour, clarity and particulates
pH	Changes to pH	Evaluation of pH
Sub-visible particulates	Evaluate particle levels over necessary range (1–100 μm)	Quantification of sub-visual particles (1–100 μm) using FlowCam
Physico-chemical analysis	Should comprise a combination of:	Techniques used include SEC, DLS, variable temperature circular dichroism, SDS electrophoresis (Tapestation) and HPLC-mass spectrometry
(a) SEC
(b) DLS
(c) CEX
(d) Capillary or SDS electrophoresis
(e) Circular dichroism
(f) FT IR
Chemical	Can comprise of: (a) HPLC	HPLC-mass spectroscopy performed on intact antibody and individual amino acid chains
(b) UV
(c) Mass spectrometry
Biological activity	Relevant to specific pharmaceutical action. Can comprise any of:	Cell based assays measuring % viability of WEHI cells
(a) Biochemical (ELISA)
(b) Cell based
(c) Animal

**Table 3:** SE-HPLC analysis of Remsima monomer concentration given as mean AUC (mAU min) over the period of the study for clinically high (1.88 mg/mL), clinically common (0.84 mg/mL) and clinically low (0.60 mg/mL) batches. Each batch contains 4 replicate devices, with each device tested in triplicate.

Day					
	0	1	2	4	7
1.88 mg/mL	103.41	103.27	102.57	103.26	103.28
100.16	99.92	99.68	100.01	99.67
103.47	102.91	102.44	102.13	102.88
105.76	105.72	105.05	104.98	105.18
Mean AUC	103.20	102.95	102.43	102.59	102.75
S.D.	2.30	2.38	2.19	2.08	2.29

Day					
	0	1	2	3	7
0.84 mg/mL	46.41	46.48	46.36	46.37	46.41
45.3	45.26	45.09	45.40	45.19
45.99	45.74	45.79	45.83	45.70
45.73	45.42	45.58	45.69	45.29
Mean AUC	45.86	45.72	45.71	45.85	45.65
S.D.	0.47	0.54	0.53	0.41	0.56

Day					
	0	1	2	4	7
0.60 mg/mL	33.03	32.74	32.48	32.64	32.81
	34.79	34.46	34.67	34.38	34.48
	32.66	32.78	32.52	32.34	32.65
	34.67	34.50	34.46	34.32	34.45
Mean AUC	33.79	33.62	33.53	33.42	33.60
S.D.	1.10	0.99	1.20	1.08	1.00

**Table 4:** Deconvoluted CD data for Remsima^®^ samples at 0.60 mg/mL, 0.84 mg/mL and 1.88 mg/mL (acquired at 76 °C) over the period of the study. All values stated are given as the percentage of total structural abundance in α-helix or β-sheets.

	Day 0		Day 1		Day 2		Day 3		Day 4		Day 7	
mg/mL	*α*	*β*	*α*	*β*	*α*	*β*	*α*	*β*	*α*	*β*	*α*	*β*
0.6	8.3	33.2	8.6	32.7	9.7	30.7			9.3	31.7	9	31.7
0.84	8.2	33.1	8.4	32.9	7.9	33.6	7.9	33.7			8	33.8
1.88	8.2	33.6	10	29.9	7.7	34.3			7.2	35.1	9.1	31.9

**Table 5:** Averaged drug abundance (percentage) of 1.88 mg/mL, 0.84 mg/mL and 0.60 mg/mL Remsima^®^ samples over the course of 7 days as determined by DLS.

	Day 0 (SEM)	Day 1 (SEM)	Day 2 (SEM)	Day 3 (SEM)	Day 4 (SEM)	Day 7 (SEM)
1.88 mg/mL	99.82 (±0.12)	99.85 (±0.03)	99.98 (±0.02)		99.92 (±0.04)	99.92 (±0.02)
0.84 mg/mL	99.52 (±0.24)	99.28 (±0.12)	99.59 (±0.05)	99.65 (±0.29)		99.77 (±0.05)
0.60 mg/mL	99.83 (±0.10)	99.93 (±0.06)	99.9 (±0.06)		99.79 (±0.09)	99.84 (±0.05)

**Table 6:** Statistical analysis of functional activity. Data shown in Fig. 8 were subjected to an unpaired *t*-test using GraphPad Prism version 5.04 for Windows (GraphPad Software, La Jolla California USA, www.graphpad.com). Day 0 was compared with Day 1 (the SPC limit) and subsequently, Day 7 with Day 1.

	Data tested	D1 vs D0	D1 vs D7
1.88 mg/mL	p-value	0.011	0.2877
Are means sig diff?	Yes	No
Summary	*	ns

	Data tested	D1 vs D0	D1 vs D7
0.6 mg/mL	p-value	0.0016	0.2777
Are means sig diff?	Yes	No
Summary	**	ns

	Data tested	D1 vs D0	D1 vs D7
0.84 mg/mL	p-value	<0.0001	0.3053
Are means sig diff?	Yes	No
Summary	****	ns

ns denotes not significant (*P* > 0.05).

* Significant (*P* ≤ 0.05).

** Very significant (*P* ≤ 0.01).

**** Extremely significant (*P* ≤ 0.0001).
