# Supplementary material for: Evaluation of the physicochemical and functional stability of diluted REMSIMA® upon extended storage—A study compliant with NHS (UK) guidance
Source: Int J Pharm. 2015 Dec 30;496(2):421–31. doi: 10.1016/j.ijpharm.2015.10.016 (PMC4687610; doi:10.1016/j.ijpharm.2015.10.016)
Supplement: Supplementary file 1 [file mmc1.doc]

***Supplemental Data***

**Evaluation of the physicochemical and functional stability of diluted *REMSIMA*® upon extended storage – A study compliant with NHS (UK) guidance.**

Benjamin L. Young ^a,†^, Monika Ali Khan ^b,†^, Terry J. Chapman ^a^, Richard Parry ^b^, Maria A. Connolly ^b^, Andrew G. Watts ^a,*^

^a^ *Department of Pharmacy & Pharmacology, University of Bath, Claverton Down, Bath BA2 7AY*

^b^ *Bath ASU, Unit A15 Fiveways Light Industrial Estate, Westwells Road, Corsham SN13 9RG*

^†^ These authors contributed equally to this work.

**1. Validation of SE-HPLC method**

1.1 **Sample preparation**

The mobile phase was 300 mM NaCl + 50 mM Na_3_PO_4_ buffer at pH 6.8 in HPLC grade water.

The injection volume was 20 μL, with an isocratic flow rate of 0.5 mL/min and UV absorbance measured at 280 nm. A needle wash solution of 10% MeOH-water was used and column temperature was ambient. A run time of 30 minutes was used for each injection.

**1.2 Demonstration of Linearity**

Linearity was evaluated at five concentration levels covering 20 - 200% of the test range. Three independent sets of standards were prepared by dilution of a standard stock solution, and analysed to construct calibration curves of AUC (mAu.min) vs concentration (mg/mL). Linearity was evaluated by regression analysis using the method of least squares. The response at each concentration level was averaged and the resulting calibration equation used to quantify drug concentration in subsequent analysis. The correlation coefficient, y -intercept and slope of the regression line were also determined and used for statistical analysis.

| **A** |  | | **Concentration (mg/mL)** | | | | |
| --- | --- | --- | --- | --- | --- | --- | --- |
|  |  | | **0.30** | **0.75** | **1.50** | **2.25** | **3.00** |
| **Peak Area (mAu.min)** | | **Calibration 1** | 18.11 | 43.83 | 85.99 | 129.31 | 171.21 |
|  |  | **Calibration 2** | 18.13 | 43.83 | 85.77 | 128.75 | 172.96 |
|  |  | **Calibration 3** | 18.05 | 44.09 | 85.87 | 128.46 | 171.97 |
|  |  | **Mean** | **18.10** | **43.91** | **85.88** | **128.84** | **172.05** |
|  |  | **SD** | 0.04 | 0.15 | 0.11 | 0.43 | 0.88 |

**B**


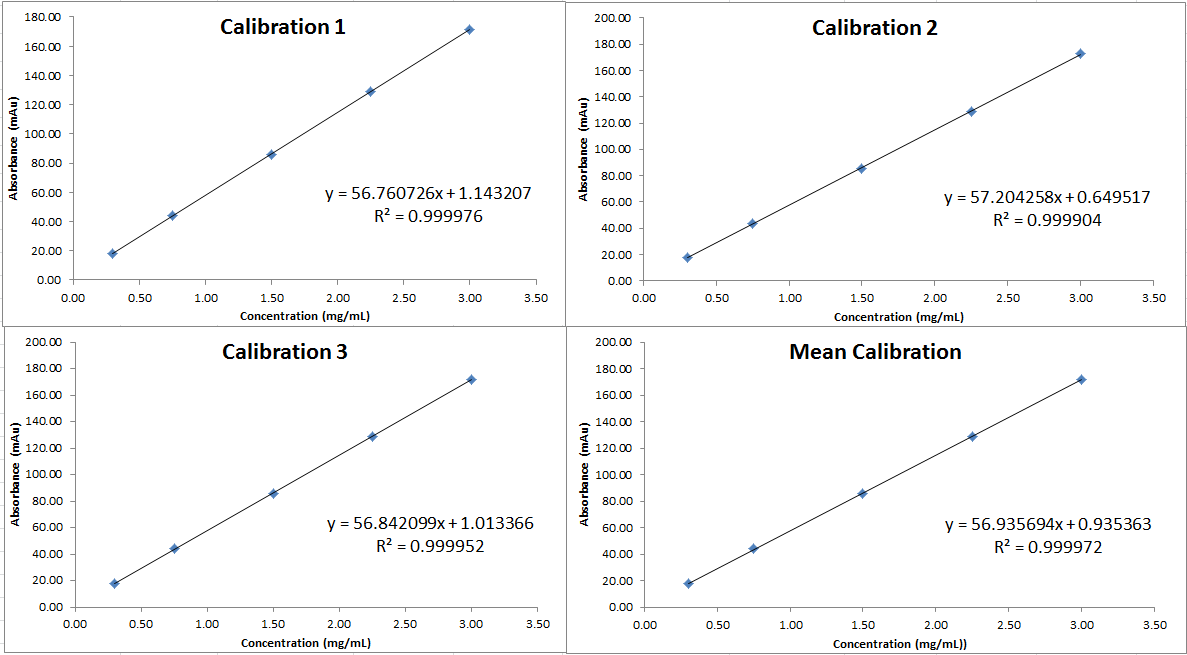


**Figure 1.** Demonstration of linearity for HPLC detection of Remisma over 20-200% of test concentrations. Drug abundance was determined by calculating the area under the curve (AUC) following HPLC analysis. Three separate analyses were performed testing remsima at five concentrations. **A**) AUC measured for each calibration over the range of concentrations. **B**) Measured AUC plotted against concentration for each calibration. The coefficient of determination (*R*^2^) values were determined by mathematical analysis of the linear regression plots.

**1.3 Determination of LOD and LOQ**

Sensitivity of the method was determined by evaluation of Remsima samples ranging from 0.3 mg/mL to 3.0 mg/mL in triplicate. Limit of detection (LOD) is the lowest amount of analyte that can be detected, but not necessarily quantified. It can be estimated from the calibration curve (Fig 1 B) using the equation LOD = 3.3 x σ ⁄ Slope. The limit of quantification (LOQ) is the lowest amount of analyte that can be quantified with acceptable accuracy and precision, and can be estimated from the calibration curve using the equation LOQ = 10 x σ ⁄ Slope, where σ is the standard deviation of y-intercepts of regression lines. Data used for the determination of LOD and LOQ is presented in Table 1.

|  | **Y-intercept** | | **Slope** | **R - Squared** | |
| --- | --- | --- | --- | --- | --- |
| **Calibration 1** | 1.1432 | | 56.7607 | 1.0000 | |
| **Calibration 2** | 0.6495 | | 57.2043 | 0.9999 | |
| **Calibration 3** | 1.0134 | | 56.8421 | 1.0000 | |
| **Mean Calibration** | 0.9354 | | 56.9357 | 1.0000 | |
| **SD** | 0.2559 | | 0.2361 |  |  |
| **LOD** | **0.01** | **mg/mL** |  |  |  |
| **LOQ** | **0.04** | **mg/mL** |  |  |  |

**Table 1.** Determination of LOD and LOQ. Both LOD and LOQ were calculated using data generated previously (Fig. 1). Slope and Y –intercept were calculated for each test, along with standard deviation of the Y-intercept values (σ).

**1.4 Precision**

The precision expresses the closeness of agreement between a series of measurements obtained from multiple sampling of a single homogenous sample under the prescribed conditions. The intra-assay-precision was established by performing six determinations at 100% of the test concentration and is reported as the % Relative Standard Deviation (Table 2).

| **Nominal Concentration (mg/mL)** | **Replicate** | **Peak Area (mAu.min)** | **Concentration (mg/mL)** | **%RSD** |
| --- | --- | --- | --- | --- |
| 1.5 | 1 | 87.32 | 1.52 |  |
|  | 2 | 86.81 | 1.51 |  |
|  | 3 | 86.80 | 1.51 | **0.47** |
|  | 4 | 86.44 | 1.50 |  |
|  | 5 | 86.44 | 1.50 |  |
|  | 6 | 86.18 | 1.50 |  |

**Table 2.** Determination of precision for HPLC analysis of Remsima samples under standard assay conditions. Intra-assay-precision was established by six determinations at 100% of the test concentration and reported as the %Relative Standard Deviation.

**1.5 Accuracy**

Determination of the closeness of agreement between the true value and reported value. This was determined by performing the method on an analyte of known purity (e.g. reference material) and was assessed by a minimum of nine determinations over three concentration levels covering the specified range. Accuracy is reported as the percentage recovery between the mean and the accepted true value (Table 3).

|  |  | **Nominal Concentration (mg/ml)** | | |  | **Nominal Concentration (mg/ml)** | | |
| --- | --- | --- | --- | --- | --- | --- | --- | --- |
| **Peak Area (mAu.min)** | **Replicate** | **0.75** | **1.50** | **2.25** |  | **0.75** | **1.50** | **2.25** |
|  | **1** | 43.03 | 86.03 | 128.26 | **Measured Conc. (mg/mL)** | 0.74 | 1.49 | 2.24 |
|  | **2** | 42.83 | 86.33 | 128.42 |  | 0.74 | 1.50 | 2.24 |
|  | **3** | 42.48 | 86.14 | 129.08 |  | 0.73 | 1.50 | 2.25 |

**Table 3.** Determination of accuracy for HPLC analysis of Remsima samples under standard assay conditions.

**1.6 Generation of a standard protein curve**

A standard curve was generated to determine retention time as a function of protein molecular weight. A protein standard mix (Fluka; PN: 69385) was run using standard assay conditions on a TSKgel© G4000SWxl column. The elution profile for this mixture (Figure 2A) provided a retention time for each protein of known mass (Figure 2B), which was used to generate a standard curve (Figure 2C).

**A B**


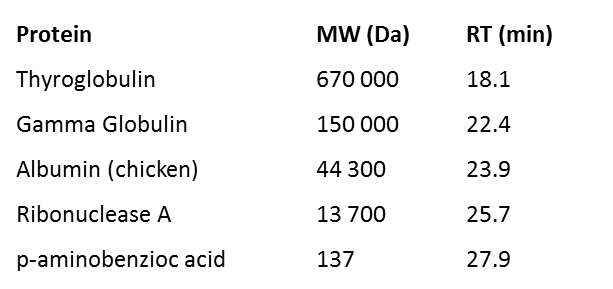


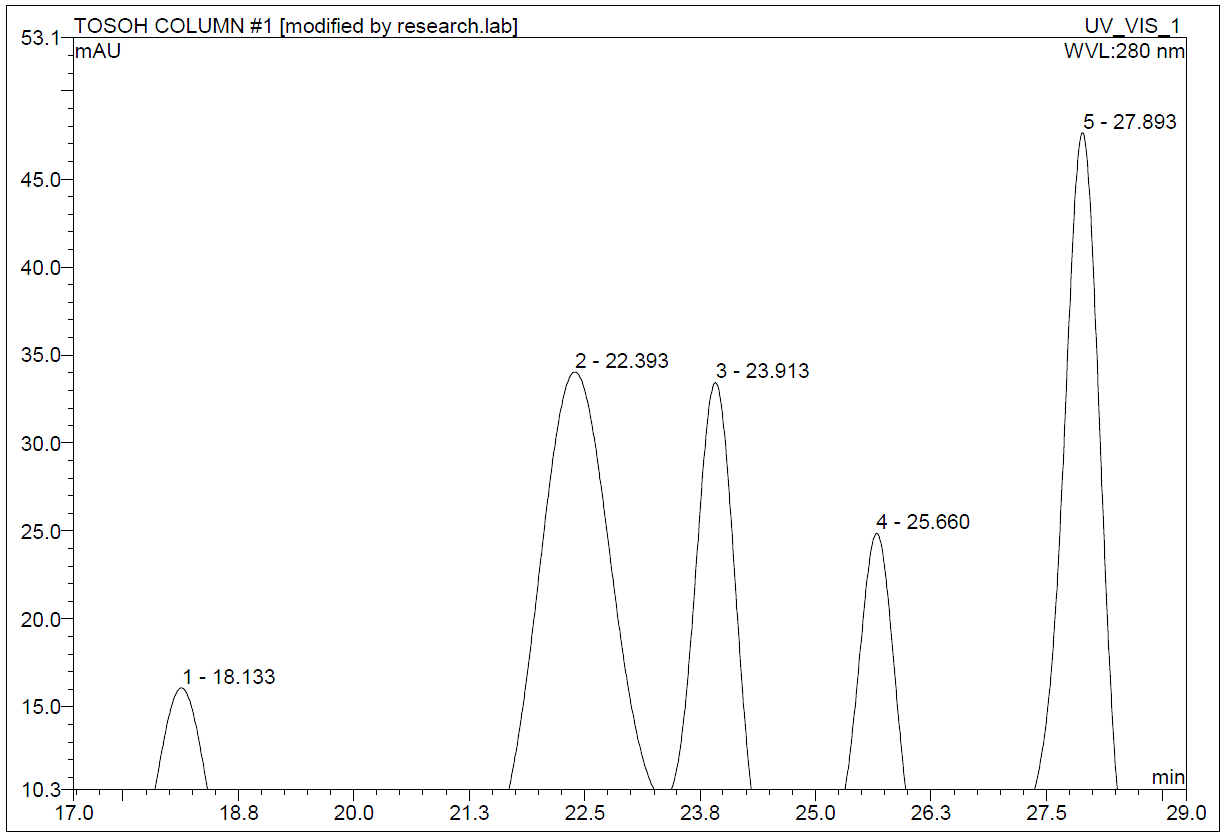


**C**

**
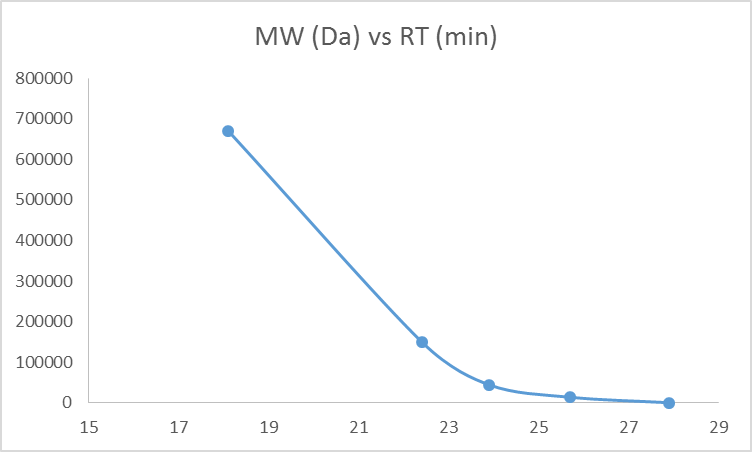
**

**Figure 2.** Determination of a standard curve for retention time (min) against molecular weight (Da). **A**) Elution profile of the protein standard mix run under assay conditions. **B**) Table of retention times observed for standard mix proteins of known molecular weight. **C**) Plot of observed retention time against molecular weight. Curve is essentially linear over a MW range of 50 – 670 kDa.

**2. Forced Degradation studies**

To demonstrate the stability indicating nature of each analytical technique, samples of freshly prepared Remsima (1.5 mg/mL) were subjected to the following stress conditions prior to analysis:

**Oxidative stress:** Remsima samples were subjected to oxidative stress by treatment with hydrogen peroxide (2.2%, v/v) at 40 ⁰C for 1hr. The stressed samples were analysed at 1.5mg/mL.

**Photolytic stress:** A sample Remsima (1.5mg/mL) in 0.9% saline solution was placed on a polarising box (40,000 Lux) and irradiated for 144 hrs.

**Change in pH:** For the forced degradation study at low and high pH (2.43 and 10.2) at 40⁰C after 9 min and 20 minutes respectively, resulted in deamidation of Asn and Gln residues. We have noticed a significant degradation and aggregates formations upon hydrolysis catalysed by base or acid (72% and 51% loss of the product).

**2.1 Demonstration of stability indicating nature of sub-visible particle counting**

Stressed samples of Remsima were analysed under standard conditions using the FlowCam technique to evaluate levels of sub-visible particles. Significant increases in particle numbers were clearly observed in samples subjected to all stress conditions (Table 4).


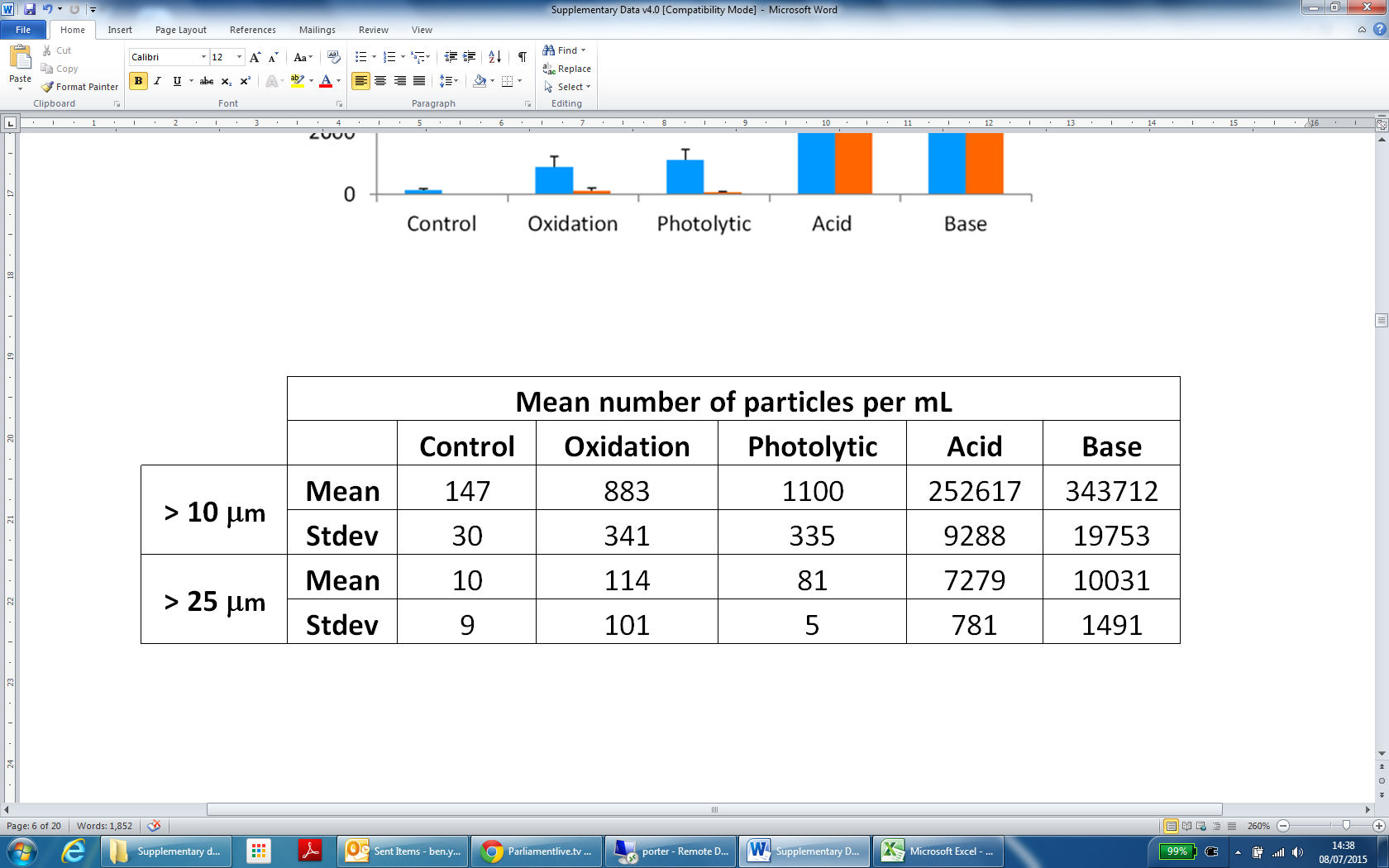


**Table 4.** Analysis of stressed Remsima samples using FlowCam technique to evaluate changes in number of sub-visible particles. Control represents a sample of fresh (unstressed) Remsima.

**2.2 Demonstration of stability indicating nature of SE-HPLC**

Stressed samples of Remsima were analysed under standard conditions using SE-HPLC technique to changes to protein size. Observable changes were clearly detected in samples subjected to all stress conditions (Figure 3).

**A B**


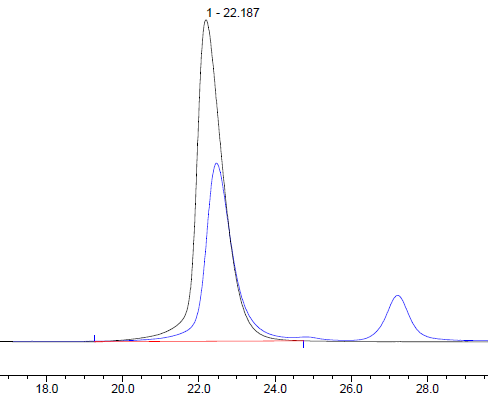

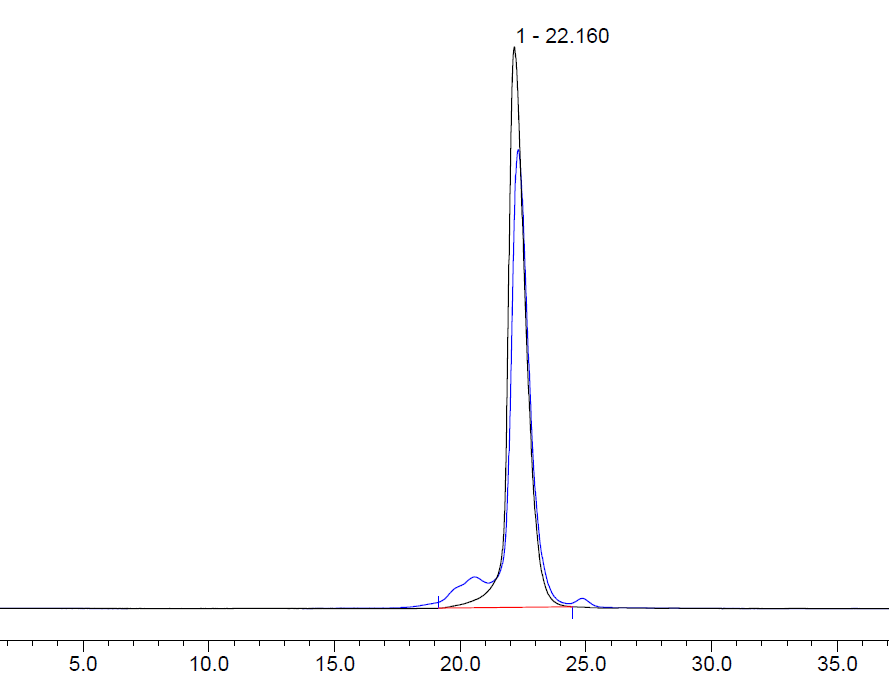


**C D**


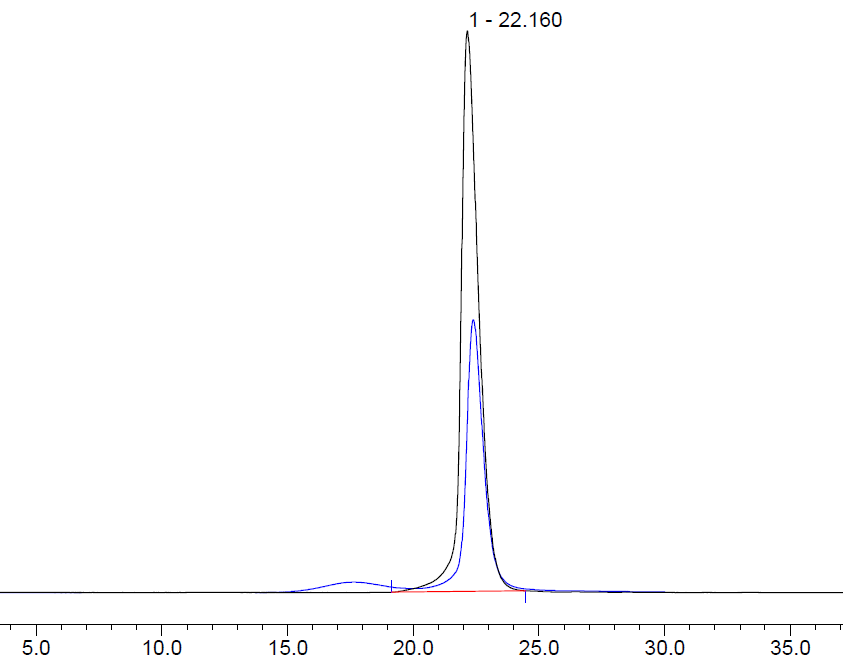

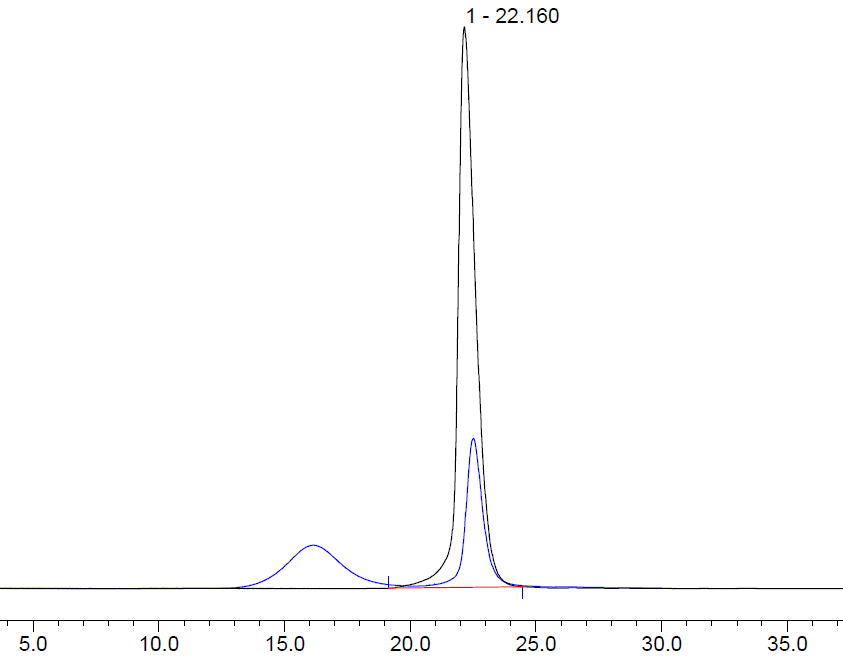


**Figure 3.** Analysis of stressed Remsima samples using SE-HPLC technique to evaluate changes in protein size. Chromatograms correspond to samples subjected to: **A**) Oxidative stress, **B**) Photolytic stress, **C**) Low pH stress, and **D**) High pH stress.

**2.3 Demonstration of stability indicating nature of Dynamic Light Scattering**

Stressed samples of Remsima were analysed under standard conditions using the Dynamic Light Scattering technique to evaluate changes to the radius and abundance of the protein. Significant changes to radius and/or abundance were clearly observed in samples subjected to all stress conditions (Table 5).

**
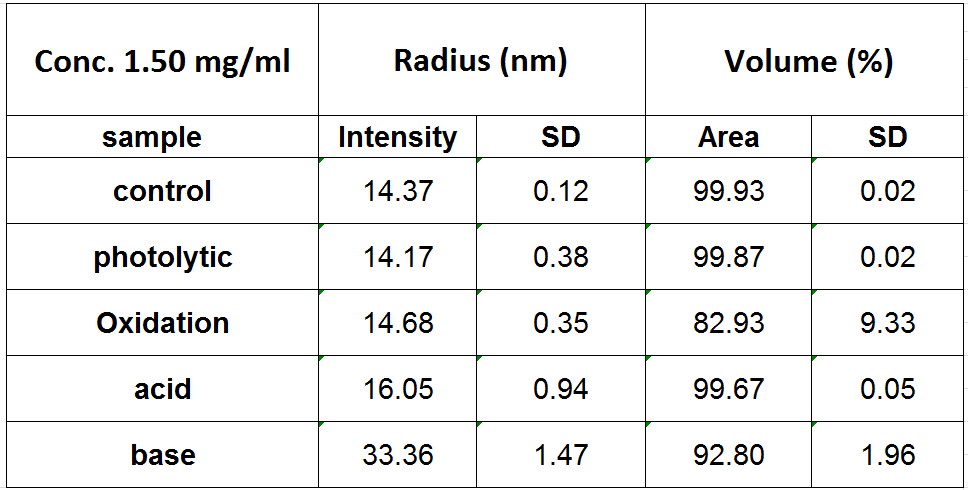
**

**Table 5.** Measured radius of protein and signal intensity by % volume for stressed samples of Remsima, as measured by dynamic light scattering.

**2.4 Demonstration of stability indicating nature of VT-Circular Dichroism**

Stressed samples of Remsima were analysed under standard conditions using the VT-Circular dichroism technique to evaluate changes to the secondary structure of the protein. Significant changes to the α-helix and β-sheet composition were clearly observed (Table 6).

| **Sample 76** ⁰**C** | **Structure** | |
| --- | --- | --- |
|  | α | β |
| **control** | 9.2 | 31.4 |
|  |  |  |
| **Photolytic** | 9.9 | 29.7 |
|  |  |  |
| **acid** | 6.0 | 37.4 |
|  |  |  |
| **base** | 4.7 | 40.9 |
|  |  |  |

**Table 6**: secondary structure of infliximab protein measured at 76 °C as the % abundance of alpha-helix (α) and anti-parallel sheets (β) by circular dichroism.

**2.5 Demonstration of stability indicating nature of LC-mass spectroscopy**

Stressed samples of Remsima were analysed under standard conditions using the VT-Circular dichroism technique to evaluate changes to the secondary structure of the protein. Significant changes to the α-helix and β-sheet composition were clearly observed (Figure 5).

**Figure 5.** LC-mass spectral analysis of Remsima samples subjected to forced stress conditions. Spectra represent control samples of **A**) light chain and **B**) heavy chain, as well as samples subjected to photolytic stress **C**) light chain and **D**) heavy chain; oxidative stress **E**) light chain and **F**) heavy chain; high pH stress **G**) light chain and **H**) heavy chain.

**2.6 Demonstration of stability indicating nature of Protein Separation Analysis (*Agilent***

***Tapestation®)***

Stressed samples of Remsima were analysed by gel electrophoresis technique using Tapestation® (Agilent). Changes were clearly visible to samples subjected to change in pH and photolytic stress (Figure 6), such the appearance of high molecular weight aggregates and lower molecular weight fragments.


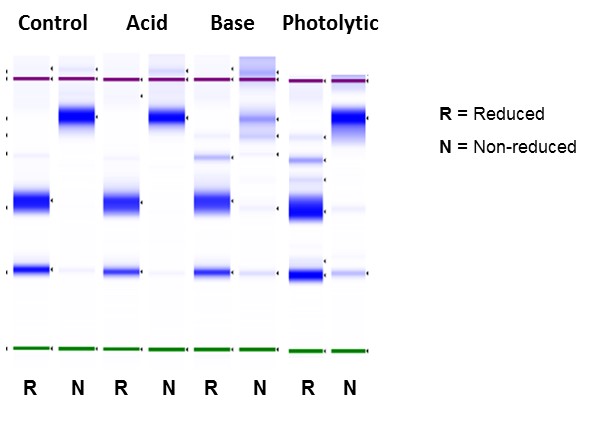


**Figure 6.** Gel-electrophoresis analysis of Remsima samples subjected to stress by change in pH and photolytic degradation.

**2.7 Method development and demonstration of stability indicating nature of biological activity assay**

Remsima biological activity was determined by its ability to bind and neutralise TNF-α in a bioassay modified from the method of Espevik et al. WEHI 164 cells are highly sensitive to TNF-α, thus in the assay cells were exposed to TNF-α in the presence or absence of remsima, and cell viability was assessed using an MTT assay.

Cell culture and stimulation: WEHI 164 cells were grown in RPMI-1640 supplemented with 10% heat inactivated foetal calf serum (FCS), and the antibiotics penicillin 100 U/mL and streptomycin 100 ug/mL. Cells were harvested from culture by rinsing with phosphate buffered saline (PBS), and incubating with 1.5 mL HyQtase. Cells were washed and re-suspended at 1.5 x 10^5^ cells/mL, and 100 uL of cell suspension added to each well of a 96 well plate, and left to adhere for two hours at 37^o^C. Dilutions of antibody samples were incubated in TNF-α 10 pg/mL for 30 minutes at 37^o^C before addition (10 μL /sample) to cells. Actinomycin D (2 µg/mL) was then added to each well and the plates incubated overnight at 37^o^C. Cell viability was determined the following day using the MTT assay.

MTT Assay: Growth medium was removed from wells and replaced with 100 uL MTT assay medium (RPMI 1640 without phenol red, 10% (v/v) FCS supplemented with 12 mM MTT). The plate was incubated for three hours at 37^o^C before removal of MTT assay medium and addition of 100 uL DMSO. Absorbance was measured at 540 nm (A540) using a Fluostar Omega plate reader (BMG Labtech). To analyse the results, the A540 of cells exposed to TNF-α in the absence of infliximab were defined as background, and these values subtracted from all samples. Cells exposed to Actinomycin D in the absence of TNF-α were set to 100%. Percent cell viability was then calculated as: % Viability = A540 sample/ A540 100% *100.

Remsima test dose for use in the bioassay was determined by preparing a dose reponse curve (figure) that demonstrated that 1 μg/ml remsima would provide a good biological response with sensitivity to loss of potency. A dose response curve was also prepared for infliximab (remicade) for comparison. After reference to the dose response curve (figure), remsima was tested in the assays at 1 µg/mL.


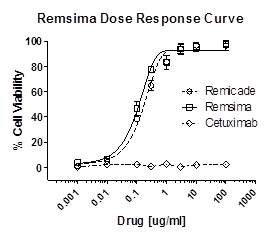


**Figure 7.** Dose response curve measuring antibody activity (% viability) against concentration (µg/mL). Activity was measured using MTT assay against Remsima (test drug), Remicade (positive control) and Cetuximab (negative control).

The stability indicating nature of the bioassay was evaluated in two stages. Firstly the assay was demonstrated to be specific for antibody binding by testing the performance of remicade (positive control) and cetuximab, an isotype (IgG1) matched, but non responsive antibody used as a negative control. Cetuximab showed no activity in this assay, whereas remicade activity was near identical to remsima (figure). Secondly, the assay sensitivity was demonstrated by evaluating the response to samples of remsima that had been confirmed as degraded by HPLC analysis were tested. Remsima samples degraded by exposure to acid or base, but not light showed loss of potency compared to intact remsima (Figure 8).**Figure 8.** Analysis of functional activity (MTT assay) of Remsima samples subject to stress conditions.

**3. Results of Visual Inspection**

Photographs of samples tested, taken at each time point. Samples **A 1-4** represent batch at 1.88 mg/mL, samples **B 1-4** represent batch at 0.60 mg/mL, and samples **C 1-4** represent batch at 0.84 mg/mL.

**
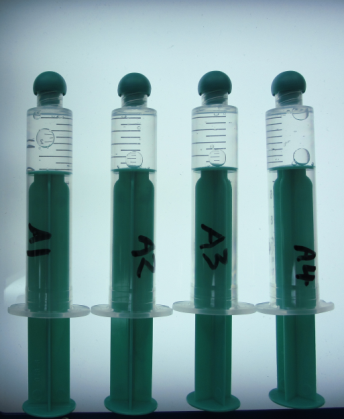

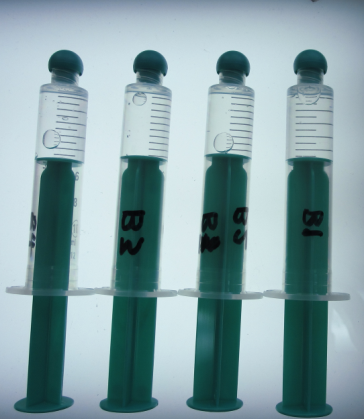

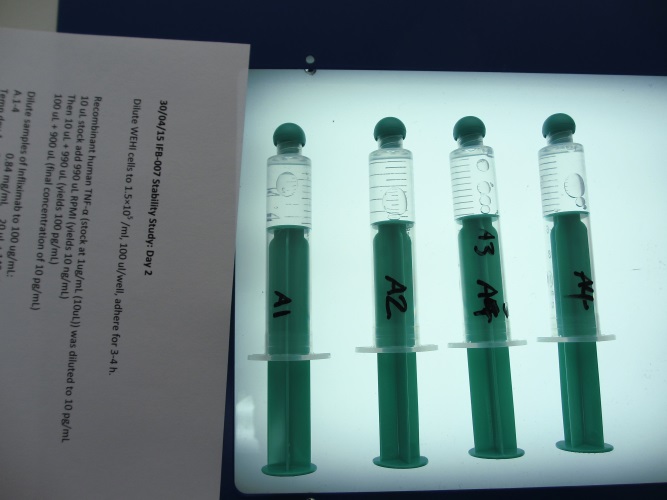
Day 2**

**A1 A2 A3 A4 B1 B2 B3 B4 C1 C2 C3 C4**

**Day 7**

**A1 A2 A3 A4 B1 B2 B3 B4 C1 C2 C3 C4**


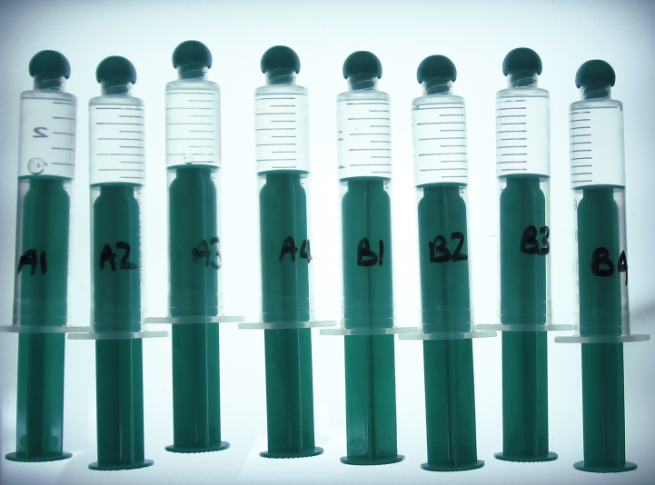

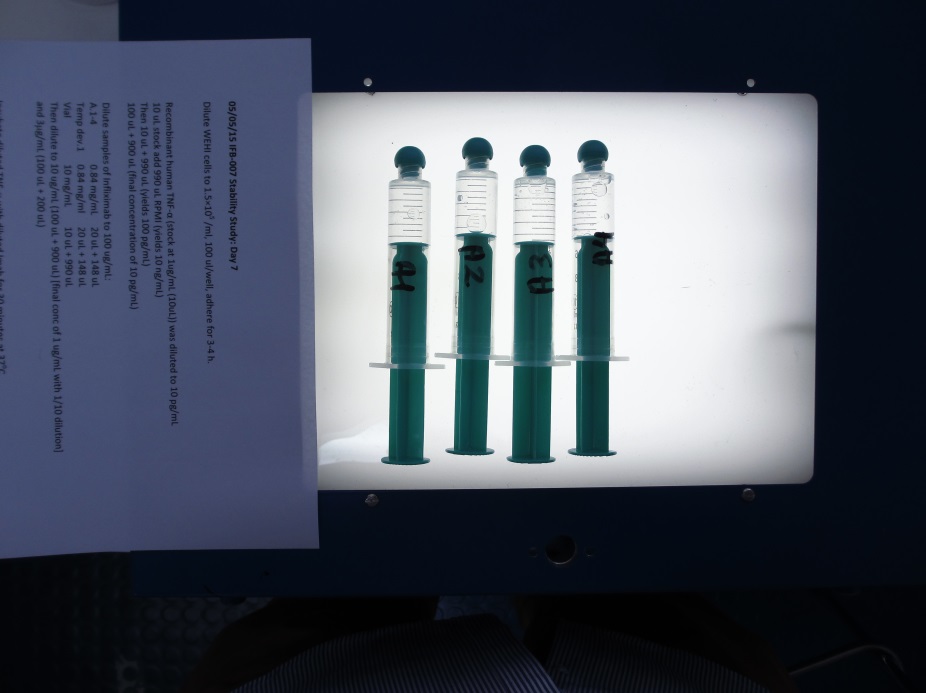


**4. Results of gel electrophoresis**

Gel electrophoresis results of samples tested, taken at each time point. Samples **A 1-4** represent batch at 1.88 mg/mL, samples **B 1-4** represent batch at 0.60 mg/mL, and samples **C 1-4** represent batch at 0.84 mg/mL.

**Day 0**


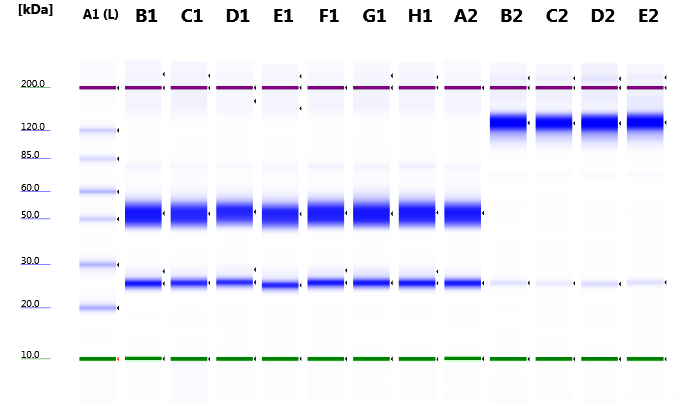

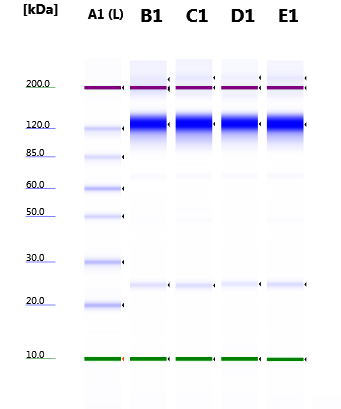

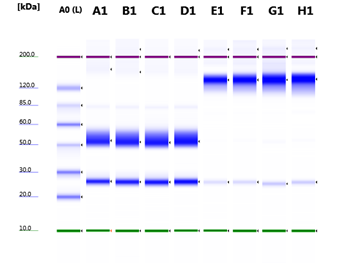


A1 A4 A3 A4 B1 B2 B3 B4 A1 A2 A3 A4 B1 B2 B3 B4

C1 C2 C3 C4 C1 C2 C3 C4

Reduced Non-reduced Reduced Non-reduced

**Day 1**


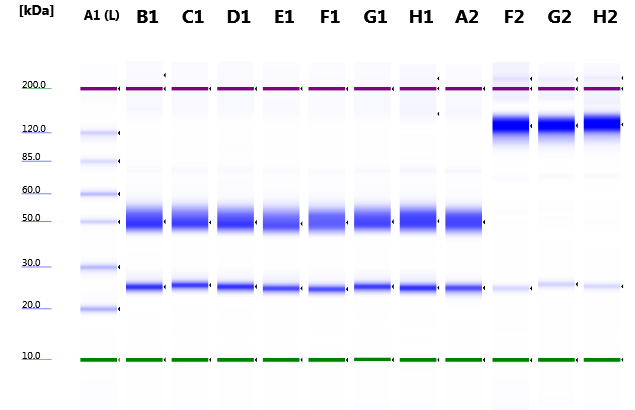

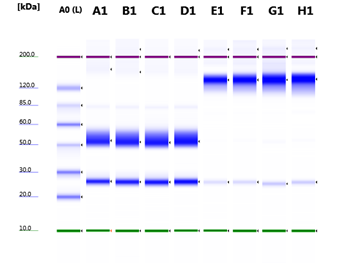


C1 C2 C3 C4 C1 C2 C3 C4


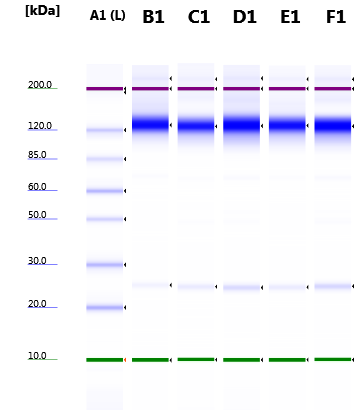


A1 A4 A3 A4 B1 B2 B3 B4 A1 A2 A3 A4 B1 B2 B3 B4

Reduced Non-reduced Reduced Non-reduced

**Day 2**


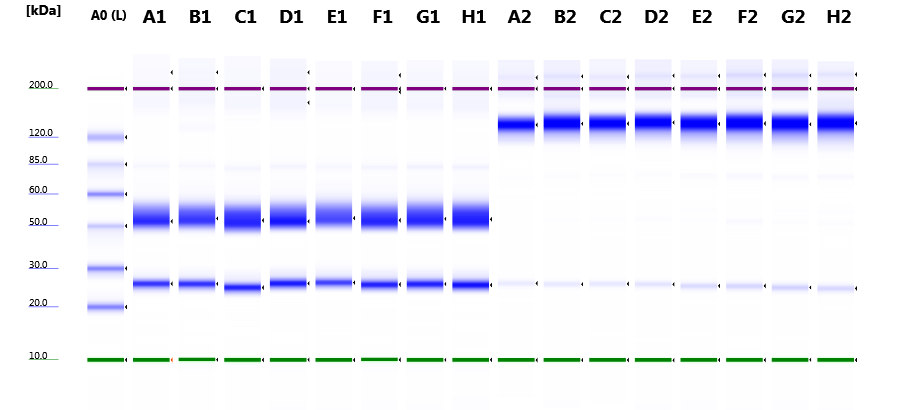

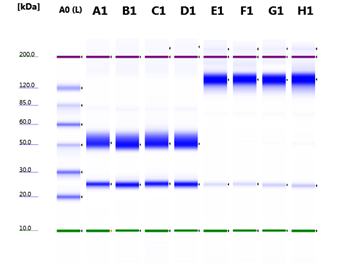


C1 C2 C3 C4 C1 C2 C3 C4

A1 A4 A3 A4 B1 B2 B3 B4 A1 A2 A3 A4 B1 B2 B3 B4

Reduced Non-reduced Reduced Non-reduced

**Day 4 Day 3**


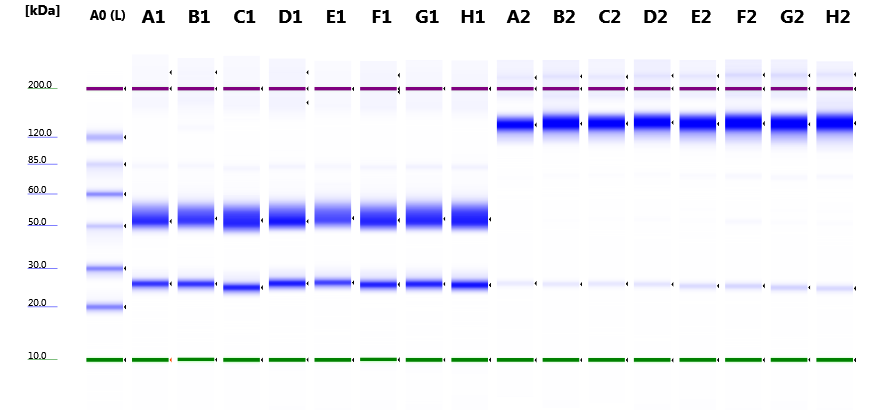


A1 A4 A3 A4 B1 B2 B3 B4 A1 A2 A3 A4 B1 B2 B3 B4


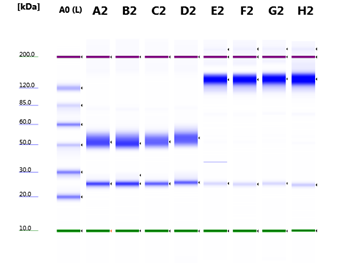


C1 C2 C3 C4 C1 C2 C3 C4

Reduced Non-reduced Reduced Non-reduced

**Day 7**


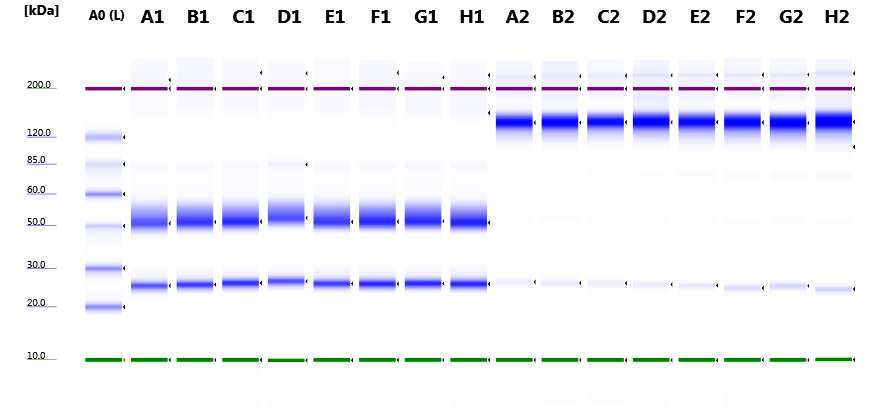


A1 A4 A3 A4 B1 B2 B3 B4 A1 A2 A3 A4 B1 B2 B3 B4


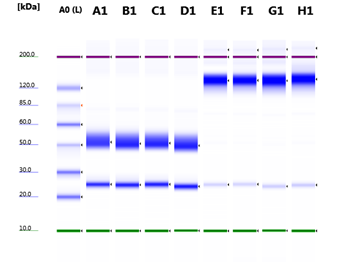


C1 C2 C3 C4 C1 C2 C3 C4

Reduced Non-reduced Reduced Non-reduced

**5. Results of LC-mass spectroscopy**

LC-mass spectroscopy results of samples tested, taken at Day 0, 1 and 7 time points. Analysis of heavy chain and light chain are presented separately for **A**) 1.88 mg/mL, **B**) 0.60 mg/mL, and **C**) 0.84 mg/mL batches.

**A)** Samples at 1.88 mg/mL

**B)** Samples at 0.60 mg/mL

**C)** Samples at 0.84 mg/mL
